# A Historical and Biographical Sketch of Geo. Watt, M.D., D.D.S.

**Published:** 1899-05

**Authors:** J. Taft


### A Historical and Biographical Sketch of Geo. Watt, M.D., D.D.S.

BY J. TAFT.

Read at a Memorial Meeting, held in the Ohio College of Dental Surgery, April 12, 1899.

In accordance with an invitation by several dentists, I here present for your consideration, a brief sketch of the life and career of the late Dr. Geo. Watt, of Xenia, Ohio.

Dr. Watt was born on the 14th of March, 1820, on a farm about eight miles east of Xenia, Greene County, Ohio. His father, Hugh Watt, was born in the north of Ireland of Scotch parentage, his mother was a native of Western Pennsylvania and also of Scotch parentage.

Mr. Watt, sons and his wife possessed the firm and sturdy character of the Scotch people. This portion of Greene County at the time that this home was established was an entirely new country. Very little had been done in the neighborhood in the way of opening and bringing under cultivation the rich land of that section. Many of the animals of the native forest were still there.

Dr. Watt related many incidents of hunting, capturing and killing wild deer, wolves, bears, catamounts, etc.

Dr. Watt's early training was under the high moral and strictly orthodox religious influence, characteristic of the Scotch and Scotch-Irish people. It was of a sturdy and full pronounced type that manifested itself all the way through the career of the subject of this sketch. His educational training was commenced at the age of seven years. The schools at that time, and in that part of the country were very primitive, and were continued but for a small portion of each year. He remained at the family home till October, 1835, when he left and went to Adams County, Ohio, and entered a boys' academy, established and conducted by Rev Wm. Taylor.

He entered upon his course in his own home engaging to pay his way, including boarding and tuition, by services; such as he could render on a farm and in connection with the school. Under this arrangement he was only able to devote a little more than one-half of his time to study, notwithstanding this disavantage, so persevering and industrious was he, that he made rapid progress.

This kind of life, away from home and its genial influence, was a new experience to him. He found an absence of home surroundings and sympathy, and the affection he had so fully enjoyed in his father's house. It was a great revelation to him. The embarrassments that came upon him and the treatment he received made a strong impression upon him, more pronounced from the fact that he was of feeble constitution, and often sick. The family with whom he was, seemed not to be in full sympathy with him and exactions were oftentimes put upon him, that he was ill able to bear, and from it he often suffered greatly; so that he was taxed much beyond his strength. At a number of times he was severely ill, and in two or three instances it was thought he was past recovery.

He remained in the academy two years, during which he devoted his time to the study of mathematics, English and the Latin language. In these he made rapid progress, he kept up with his class, the other members of which devoted all their time to study. At the end of two years he engaged in teaching in a common country school, in the vicinity of the academy, remaining there however for only about one year, when he returned to his father's home, and there engaged again in teaching. He was thus occupied for about four years. He was successful and popular as a teacher.

In 1840 he entered a college at Ripley, Brown County, Ohio, remaining there about one year, leaving there in 1841 and returning to Greene County and there again engaged in teaching; and began the study of medicine under the supervision of Dr. Samuel Martin, at that time a noted and successful physician of that place. In Dr. Martin he found an instructor with whom he not only had superior instruction in medical science and practice, but he also had there inculcated in the receptive mind, the views of the dignity and importance of medical science and practice, that chracterized him throughout his life.

He practiced with and for his preceptor about one year after which he removed to Bentonville, a small town in Fayette County, Indiana, where he soon established an excellent practice, especially for a new country.

Not being satisfied with his attainments in medical science, he entered the Medical College of Ohio in 1846.

He graduated with honor in March, 1848.

He was so fortunate as to take his medical course under the instruction of a faculty whose professors were shining lights in the medical profession, embracing the celebrated R. D. Mussey, who, as a surgeon, had no superior in this country; J. T. Shotwell, that celebrated anatomist; Dr. Harrison, Professor of Theory and Practice, a physician of world-wide reputation, and Dr. J. Locke, Professor of Chemistry, who, as a teacher of this branch at that day, had no superior.

Dr. Watt enjoyed the high privilege of especial acquaintance of each of these gentlemen, and such an acquaintance as insured to him the very best that these professional men could give to an industrious, capable, eager and enthusiastic student. The privileges thus enjoyed were utilized to their full extent by Dr. Watt, and to this fact, may in large measure, be attributed the high order of medical attainments which he enjoyed throughout his life.

Dr. Watt possessed a very high estimate of the dignity of the medical profession, as such he was intolerant to quackery and shams of all sorts in his profession.

On April 17, 1845, he was married to Miss Sarah Jane McConnell, who was a native of Greene County, and had always resided with her father's family near Xenia. Like the subject of this sketch, she was the youngest of a large family. These two families were for a long time neighbors, and intimate friends, indeed they journeyed together in coming to Ohio.

After the completion of his medical course he practiced his profession in Indiana about one year.

Near the close of 1848 Mrs. Watt was poisoned by some means, never fully explained, whether by accident or by some evil-disposed person was never ascertained. The case came well nigh being fatal, recovery from it was slow, and the result as to recovery was for a time doubtful. This condition of things made it necessary for the Doctor to leave his practice and return to the vicinity of his former home.

This change brought him to the practice of medicine in Xenia, where he remained till the spring of 1850, when he removed to Kenton, Ohio, where he continued in practice about two years. About this time his love for, and inclination to special scientific pursuits, began to be rapidly developed, as was shown by various investigations in which he engaged.

Early in 1852 he entered upon the study of dentistry, for which a good foundation had been laid, in his very thorough medical course. It is very rare indeed that any one has the same preparation for the course in dentistry, as that possessed by Dr. Watt. His early medical education was superior. He practiced medicine for a time, then took a regular medical course, for all this work he possessed a thorough mental qualification as well as inclination.

His attainments in chemical science were of a very high order. Having a love and enthusiasm for it, with the highest order of instruction, he made unusual attainments in this branch of science. He began his preparation for this specialty with the writer of this sketch, and it was not long after this, till it was quite apparent, that in the knowledge of the principles underlying the dental practice, the presumed teacher found his pupil quite his superior, but the knowledge of technic and practical matters by the teacher was set over against the superior knowledge of principals by the pupil, each was a pupil and each an instructor, so that there was no occasion for assumed superiority on part of either, at least this was the view entertained by both parties, so that all things went along harmoniously.

After about a year devoted to this study a partnership was formed between the teacher and pupil; this relationship was continued for many years, a year or two in Xenia, and subsequently in Cincinnati.

Dr. Watt's ability as a teacher and as a thorough chemist was so well recognized that in 1853 he prepared and delivered a course of lectures on Chemistry, in the Ohio College of Dental Surgery. Dr. Elijah Slack, L.L.D., had delivered a course of lectures on Chemistry in this same institution for one or two years before, yet Dr. Watt made the first attempt to adapt a course of lectures on Chemistry to the needs of the dental student, and he was the first to deliver such a systematic course. Hitherto, all the work in this line had simply been such as was given to the medical students. During the time in which this course was delivered, he was not only a teacher, but was arranging a new course for himself, and was a member of the class, so that he was in the double capacity of a teacher and a pupil.

He graduated with his class, receiving the degree of D.D.S. at the close of the term of 1854.

Though he was a graduate of medicine, with quite an extensive experience in practice and had not given more than one year to the study of dentistry and was a teacher, yet at the time of his graduation, he passed the same examination as the other members of the class.

Immediately after this he resumed the practice of dentistry with his former partner. So well recognized was his ability as a teacher, that he was not long permitted to enjoy the quiet of a town and country practice. In 1855 he was elected Professor of Chemistry and Metallurgy in the Ohio College of Dental Surgery, which position he occupied for several years, and the results, achieved by him, during this time, in the development of the application of chemistry to dental science and art, were of a very high order, and served as a stimulus for the establishment of a chair for this branch in other dental colleges, none of which had made this a special department up to this period. Though chemistry has made great and rapid progress and development during the last half century, it may be well questioned here whether the teaching of this branch in dental colleges is relatively any better to-day than that done by Dr. Watt. He was the pioneer in directing the attention of the profession to chemistry, as one of the chief subjects in dental science and art.

In 1852 Dr. Watt became a member of the Mississippi Valley Dental Society. During the many years of his membership he was always active in the interests of that organization. In 1854 a prize of $100 was offered for the best published paper on Dental Surgery. He was a competitor for this prize, with the rusult that after the examination of many other papers, the prize was unanimously awarded to him. He was for several years secretary of the body and was at the conclusion of this service elected its president. In 1856 he was a member the "*American Dental Convention*" held at Hope Chapel, in New

York. This was the largest dental organization in the world at that time. At that meeting he read a paper on "Topical Remedies," which was extensively published and elicited much attention and discussion. The article was published in the 10th volume of the DENTAL REGISTER.

In October, 1855, Dr. Watt, with the writer of this sketch, became the owners and editors of the "*Dental Register of the West*;" this at that time was the only dental journal in the West and was the second oldest in the world. This relation was maintained for many years and only ceased when Dr. Watt's failing health rendered it imperative, but he was after that a regular contributor to the "*Register*" and the profession.

He, in connection with Drs. Taft and Hammel, established an office for dental practice in Cincinnati in 1855. This relationship was continued for about three years, when the sudden death of Dr. Watt's father made it necessary for him to remove to Xenia, Ohio, where he resumed the practice that had been established many years before. About this time he made some experiments in micro-photography that were in advance of anything that had been accomplished up to that time. In 1860, in the time of the great trial of our nation, he promptly tendered his services. He was accordingly made surgeon of the 154th Regiment of the Ohio Volunteer Infantry. His usefulness and efficiency in his position will be well understood, when it is stated that the sanitary record of his charge was better than that of any other Ohio regiment. He was mustered out in September, 1864, after being disabled by an injury of the spine, having been crushed by a falling wagon, which resulted in locomotor ataxia. After his return from the army, he entered into practice as his feeble health would permit. In the summer of 1865 while in attendance upon the meeting of the American Dental Association, held at Chicago, he was severely attacked by disease which developed into cerebro-spinal meningitis. This condition was doubtless somewhat induced by his enfeebled condition already mentioned. With this severe affection he was confined to his bed for many weeks, much of the time in a semi-conscious condition, and there was but the slightest hope that he would recover. After some five or six weeks he began to show signs of improvement, and within two months the disease passed away, but left him in a very feeble and precarious condition. Because of his impaired health, he was subject to frequent attacks of various diseases; his lungs from boyhood were never strong, and were the seat oftentimes of severe affection.

The frequent and severe attacks on his lungs, without fatal results, were the occasion of surprise, and many a time astonishment, to his friends and physicians who attended him. Doubtless the resistence he was able to make to these attacks, was due, in a large measure, not so much to the skill of his attending physician, as to his own knowledge of the disease and its management. In 1865 he formed a partnership with Dr. N. W. Williams; they conducted a practice in Xenia, Ohio, for about one year, when they established a branch office in Cincinnati, of which Dr. Watt took charge. In 1868 the firm of Watt & Williams purchased the dental depot of S. J. Walters & Co., of Cincinnati. This they conducted successfully, in addition to dental practice, for about three years, when they sold the depot to Spencer & Moore.

During his last residence in Cincinnati, his strength gradually became much impaired and after disposing of his commercial enterprise and resigned his position in the college, where he had been teaching for several years, he removed to Xenia on September 30, 1871. Here he hoped to have respite from active labor, but his active brain would not permit much rest, and in the spring of 1872 the firm of Watt & Williams was dissolved. Dr. Williams going to Europe. After this Dr. Watt formed a partnership with Dr. D. G. French, which continued for about one year, at the end of which he formed a partnership with Dr. E. G. Betty, of Cincinnati. This relationship was also continued for about one year. After this he associated with him Dr. W. H. Sillito, this continued for about three years, during which time his health was becoming more feeble and his strength well nigh gone.

It was deemed best that he should retire and no longer attempt the active duties of practice, but he could not be idle, and in 1877 he assumed the editorship of the *Ohio Journal of Dental*

*Science*, a monthly journal of about fifty pages, and what it was during his administration, we will not describe here, suffice to say, it soon became recognized as one of the most valuable publications in the interest of the profession. Its pages always filled with matters of interest and profit, and much of this matter was from his facile pen. The editorials were always rich in thought and characteristic of the man, even in his best days.

In 1867 he published a volume, "Watt's Chemical Essays," which contained the principal papers which he had written on dental chemistry. Many of these papers have been published in various journals, not only of the dental, but of the medical profession as well, and even in some of the leading newspapers.

Unfortunately, the syllabus of his series of lectures on chemistry have never been published, and only remain in the memory of some of his devoted students.

He has occupied a number of positions of prominence, in addition to those mentioned. He was Vice-President of the American Dental Convention, he was elected President of the American Dental Association on the same day he became a member, the only instance of the kind on record. He was President of the Ohio State Dental Society the first two years of its existence; he was twice President of the Mad River Dental Society. In every responsible position he ever occupied the duties were discharged faithfully and efficiently.

Dr. Watt was one of the most easy, fluent and correct writers in the dental profession. His literary attainments were formed upon a high model, and is all that we could expect of one of high literary and classical culture. In his case it was inborn, rather than acquired, though, as some explanation, it may be said that his reading in early life was confined to the purest and best of English literature.

Though he lived and acted in the profession at a time of its formative period and doubtless did much to elevate to a higher position, during his career, many principles were studied and brought to a higher degree of efficiency by his efforts. His aim was ever for the betterment and elevation of his chosen profession.

Many appliances and modes of practice might, did time permit, be cited here, in which he much improved these, and in many instances devised altogether new things. A record of many of these has never been made. In reference to this it may be said, that he was modest, never anxious to put himself forward as an inventor.

Perhaps the greatest work he ever did was in the elaboration of the principles involved in "dental decay." That which he accomplished in this respect was far in advance of anything that had been previously done, and though extensive investigations and attainments have been made since the promulgation of his views and in certain directions advances made, yet some of his positions were so firmly established that they have never as yet been successfully assailed. He attained a national, and indeed an international, reputation in the profession, in which he has left an enviable heritage to the future generations of dental practitioners.

